# Nutritional Intervention for Developmental Brain Damage: Effects of Lactoferrin Supplementation in Hypocaloric Induced Intrauterine Growth Restriction Rat Pups

**DOI:** 10.3389/fendo.2019.00046

**Published:** 2019-02-08

**Authors:** Yohan van de Looij, Camille Larpin, Jan-Harry Cabungcal, Eduardo F. Sanches, Audrey Toulotte, Kim Q. Do, Stéphane V. Sizonenko

**Affiliations:** ^1^Division of Child Development and Growth, Department of Pediatrics, School of Medicine, University of Geneva, Geneva, Switzerland; ^2^Laboratory for Functional and Metabolic Imaging, Ecole Polytechnique Fédérale de Lausanne, Lausanne, Switzerland; ^3^Department of Psychiatry, Centre for Psychiatric Neuroscience, Lausanne University Hospital, Lausanne, Switzerland

**Keywords:** intrauterine growth restriction, caloric restriction, lactoferrin, neuroprotection, magnetic resonance imaging

## Abstract

**Introduction:** Intrauterine Growth Restriction (IUGR) refers to an impaired development of the fetus and hence results in adverse neurodevelopmental and psychiatric consequences later in life. Lactoferrin (Lf) is a glycoprotein present in milk that has already shown neuroprotective effects through its anti-inflammatory and antioxidant properties on impaired developing brains. The aim of this study was to characterize a rat model of IUGR and assess the neuroprotective effect of a nutritional supplementation with bovine Lf during pregnancy and lactation on this model.

**Methods:** A model of 50% gestational caloric restriction (CR) was used. Three groups were designed, and pregnant rats had either *ad libitum* access to food (control group, CTL) or 50% of the controls' intake (restricted group, IUGR). The diet was isocaloric and supplemented with bovine Lf for the caloric restricted dams (restricted-Lf, IUGR_Lf). At postnatal day 7 and 21, advanced *ex-vivo* diffusion MRI techniques at 9.4T were used to investigate brain cortical and white matter microstructure. Further, genes and proteins involved in structure (synaptophysin, MBP), microglia (Iba-1), metabolism (MCT2, βCaMKII) and apoptosis (Bcl-2) were analyzed in the cortex and striatum. In the cortex, the number of parvalbumin immunoreactive interneurons and their perineuronal nets were quantified. Behavioral tests were performed at P31.

**Results:** Effects of the CR were significant in the cortex and striatum with reduction of synaptophysin (marker of synaptogenesis) at P7 and MBP (marker of myelin) at P21 in the cortex. Indeed, MCT2 (energy metabolism), Bcl-2 (anti-apoptotic protein) and βCaMKII (synapse activity) expressions were reduced in IUGR groups at P7. In the striatum NG2 (marker of oligodendrocyte precursor cells) and Bcl-2 at P7 as well as βCaMKII at P21 were decreased following IUGR and restored by Lf. Cortical microstructure was impaired following CR with partial effect of Lf. Lf prevented oxidative stress induced parvalbumin interneurons impairments whereas striatum and external capsule showed alterations in microstructure depicted by diffusion MRI, which were also partially reversed by Lf.

**Discussion and Conclusion:** The model of 50% caloric restriction induced mild impairment partially reversed by nutritional intervention using Lf during pregnancy and lactation.

## Highlights

- Moderate undernutrition induced IUGR demonstrated mild cerebral impairment.
- Cerebral impairment is characterized by white and gray matters damage depicted by MRI.
- Oral Lf supplementation showed partial neuroprotection following caloric restriction during gestation.
- It represents a promising approach to reduce developmental diseases in IUGR and preterm infants.

## Introduction

The definition of intrauterine growth retardation (IUGR) given by world health organization in 1995 was “An infant suffering from IUGR is defined as being below the 10% percentile of the recommended gender-specific birthweight for gestational age reference curves.” It is one of the main causes of developmental brain damage with possible long life neurodevelopmental disabilities. If IUGR is an important public health concern worldwide, the rate of IUGR in the developing countries is about six times higher than in developed countries with at least 30 million IUGR infants per year. Poor antenatal and postnatal nutrition further prevents infants from attaining their full developmental potential. IUGR adults show a higher risk of altered cognitive and executive function and emotion control. The often-associated morbidities of IUGR, prematurity and poor postnatal growth represent a major risk for poor development of affective cognition and psychiatric disorders (1–3).

Lactoferrin (Lf), an iron-binding glycoprotein is involved in the intestinal iron uptake and regulates immune response with anti-inflammatory as well as antioxidant property (4, 5). In humans, higher concentration of Lf is found in the colostrum but Lf level decreases through lactation period (6). After oral administration, Lf is regulated through binding sites on brain endothelial cells allowing the passage from blood to tissues including the brain (7). Lf has been shown to down-regulate inflammation in lipopolysaccharides injury models (8). Importantly, Lf nutritional supplement in preterm shows significant reduction in late-onset sepsis and necrotizing enterocolitis (9). Our previous studies explored the effect of Lf on various animal models of perinatal brain injuries. Hypoxic-Ischemic post-natal day 3 (P3) pup rats demonstrated recovery with maternal Lf supplementation during lactation (10), characterized by a reduced cortical loss and an altered white matter recovery. Furthermore, inflammatory and apoptotic gene expressions were also reduced (10). Following lipopolysaccharides injection at P3, Lf administration during lactation reduced ventriculomegaly as well as impaired microstructure and white matter alterations (11). Finally, in a model of dexamethasone induced IUGR mimicking maternal stress and fetal growth restriction, the abnormal levels of brain metabolites as well as major neurotrophic factor (BDNF) were restored with Lf (12). Taken together, these findings suggest that Lf, with its anti-inflammatory and antioxidant properties, has great potential to reduce brain injury, improve cerebral development and prevent noxious programming due to adverse pre and postnatal conditions.

As such, the aim of this study was to evaluate structural and functional cerebral impairments in gestational caloric restriction induced IUGR as well as the potential neuroprotective effect of Lf supplementation. Effects of caloric restriction were assessed with advanced diffusion magnetic resonance imaging techniques [diffusion tensor imaging (DTI) as well as neurite orientation dispersion and density imaging (NODDI)] in conjunction with protein and gene expressions and behavioral tests.

## Materials and Methods

### Animal Preparation

All experimental protocols have been previously approved by the Animal Research Ethics of Geneva, Switzerland (#GE7115). Nulliparous Wistar female (225–250 g) and Wistar male (250–275 g) rats (Charles River Laboratories, France) were used for breeding. During gestation, they had either *ad libitum* access to food: Control group (CTL) or access to 50% of the controls' intake: Restricted group (IUGR). Bovine lactoferrin (Lf) (Taradon laboratory, Tubize, Belgium) at an expected dose of 1g/kg/day according to daily intake of food by gestating and lactating dams, was added to the diet as a supplementation also during gestation and lactation for the Restricted-Lactoferrin group (IUGR_Lf). From the day before the parturition and during the whole lactation, all dams had *ad libitum* access to food, with either Lf enriched (1 g/kg/day) or isocaloric diet. Standard animal housing conditions according to the animal facility of the CMU, University of Geneva, were applied with free access to water. At birth, rat pups were culled or boarded out to another dam of the same group in order to control litter size to 10 to 12 pups per dam until weaning day. Dam and pup weight gain were measured daily until weaning and once a week until P42. The number of pups per group is mentioned by experiment separately. The number of dams used in the study was: 6 controls, 10 IUGR and 9 IUGR_Lf.

### Brain Collection for *ex-vivo* MRI and Immunofluorescence

After intraperitoneal injection of pentobarbital (50 μg/kg), rat pups were intra-cardially perfused with 0.9% NaCl and 4% paraformaldehyde solution at postnatal day 7 (P7) and 21 (P21). Brains were removed and immersed in 4% paraformaldehyde overnight for post fixation.

### Magnetic Resonance (MR) Experiments

All MR experiments were performed on an actively-shielded 9.4T/31 cm magnet (Varian/Magnex) equipped with 12-cm gradient coils (400 mT/m, 120 μs). *Ex-vivo* MRI was performed on P7 (CTL; *n* = 6, IUGR; *n* = 6, and IUGR_Lf; *n* = 6) and P21 (CTL; *n* = 7, IUGR; *n* = 4, and IUGR_Lf; *n* = 4) fixed brains with a 2.5 mm diameter birdcage coil. A multi-b-value shell protocol was acquired using a spin-echo sequence with a field-of-view equal to 21 × 16 mm^2^ and a matrix size of 128 × 92. Twelve slices of 0.6 mm thickness were acquired with 3 averages and TE/TR = 45/2,000 ms. Ninety-six diffusion weighted images were acquired including 15 b_0_ images and 81 images separated in 3 non-collinear shells with a uniform distribution in each shell. Distribution of number of directions and *b*-value were as follow: 21 directions at a *b*-value of 1,750 s/mm^2^, 30 directions at a *b*-value of 3,400 s/mm^2^ and 30 directions at a *b*-value of 5,100 s/mm^2^. NODDI toolbox (13) was used to fit the acquired data. Regions of interest were delineated in cortex (Cx), external capsule (EC), and striatum (St). DTI derived parameters [Axial diffusivity (AD), Radial diffusivity (RD), Mean diffusivity (MD) and Fractional anisotropy (FA)] as well as NODDI derived parameters [intra-neurite volume faction (*f*_*icvf*_), isotropic volume fraction (*f*_*iso*_) and orientation dispersion index (ODI)] were averaged in the different regions assessed as previously used in animal models of perinatal brain injuries (11, 14).

### Tissue Collection for Protein and Gene Expression

Rat pups were euthanized by decapitation at P7 and P21. Pups were anesthetized with pentobarbital (50 μg/kg) prior to decapitation. Brains were quickly removed, dissected on ice and frozen instantly in liquid nitrogen. Two structures were dissected: cortex (Cx) and striatum (S). The samples were stored at −80°C until analyses.

### Immunoblotting

Brain tissues from P7 (CTL; *n* = 6, IUGR; *n* = 6 and IUGR_Lf; *n* = 7) and P21 (CTL; *n* = 10, IUGR; *n* = 14 and IUGR_Lf; *n* = 13) rats were homogenized by sonication in RIPA buffer (Cell Signaling, 9806S), and the protein concentration was assessed using a Bradford assay. Proteins (25 μg) were separated by SDS-PAGE, transferred to a nitrocellulose membrane and analyzed by immunoblotting. All antibodies (Table 1) were diluted in blocking buffer containing 0.1% casein (Sigma-Aldrich, C8654). Antibodies were diluted in the concentrations suggested on the data sheet by the manufacturer (Table 1). After incubation of the primary antibodies, the following secondary antibodies were applied: goat anti-mouse IgG (H+L) conjugated with Dylight^TM^ 680 (#5470, Cell Signaling Technology), goat anti-rabbit IgG conjugated with IRDye 800 (926-32210, LI-COR) and donkey anti-goat IgG conjugated with IRDye 680 (926-68074, LI-COR). The Odyssey Infra-red Imaging system (LI-COR) was used to visualize the protein bands. The densitometry was assessed by normalizing the optical density of each sample with respect to actin expression (mouse monoclonal anti-actin from Millipore, MAB1501), using Image Studio Lite ver.5.2 software. The results are expressed as a percentage of values obtained either for the control or for the restricted rat pups (100%) using actin as a standard.

**Table 1:** Primary antibodies for immunoblotting including catalog numbers, companies, and dilutions.

**Abbreviations**	**Primary antibodies**	**Catalog number/Company**	**Dilution**
Actin	Anti-actin antibody clone C4	MAB1501/Millipore	1/1,000
CD68	Rabbit polyclonal anti-CD68	250594/Abbiotec^TM^	1/500
DCX	Rabbit polyclonal anti-DCX	ab18723/Abcam	1/1,000
GFAP	Mouse monoclonal anti-GFAP	G6171/Sigma-Aldrich	1/1,000
Iba-1	Goat polyclonal anti-Iba1	ab5076/Abcam	1/1,000
MBP	Mouse monoclonal anti-MBP	ab62631/Abcam	1/1,000
NeuN	Mouse monoclonal anti-NeuN	MAB377/Millipore	1/1,000
NG2	Rabbit polyclonal anti-NG2	ab129051/Abcam	1/1,000
Synaptophysin	Mouse monoclonal anti-synaptophysin	ab8049/Abcam	1/500
βCaMKII	Rabbit polyclonal anti-CAMKII beta	ab34703/Abcam	1/1,000
DMT1	Rabbit polyclonal anti-DMT1	ab140977/Abcam	1/1,000
GLT1	Guinea pig polyclonal anti- GLT1	AB1783/Millipore	1/500
Leptin receptor	Rabbit polyclonal anti-Leptin Receptor	ab5593/Abcam	1/1,000
MCT2	Rabbit polyclonal anti-MCT2	AB3542/Millipore	1/1,000
NMDAR	Rabbit polyclonal anti-NMDA R2A	ab14596/Abcam	1/1,000
IGF 2	Rabbit polyclonal anti-IGF2	ab9574/Abcam	0.2 μg/mL
TrkB	Rabbit polyclonal anti-TrkB	ab18987/Abcam	1/1,000
Fractin	Rabbit polyclonal anti-Fractin	AB3150/Millipore	1/1,000

### Real-Time Quantitative PCR (RT-qPCR)

The extraction of the total mRNA was done using the RNase Mini Kit (Qiagen, 74104) following the manufacturer's instructions (Table 2). On P7 collected brains, 3 μg mRNA from the three regions of interest at P7 (CTL; *n* = 6, IUGR; *n* = 6 and IUGR_Lf; *n* = 6) and P21 (CTL; *n* = 6, IUGR; *n* = 8 and IUGR_Lf; *n* = 8) were reverse transcripted to cDNA using 400 units of Moloney murine leukemia virus reverse transcriptase (Invitrogen, 28025-013), 20 units of recombinant RNAsin (Promega, N2515), 0.5 μg of random hexamers (ThermoFischer Scientific, #S0142), 2 mmol/L dNTP (Invitrogen, 10297018), and 40 mmol/L of dithiothreitol (Invitrogen, 18080093). Real-time quantitative PCR was performed with the PowerUp SYBR Green Master Mix (Applied Biosystems, A25742) and using an StepOnePlus™Real-Time PCR System (Applied Biosystems). Gene expressions were normalized using the housekeeping ribosomal gene RPS29. The results were calculated using the Livak approach and are expressed in arbitrary units (A.U) (12).

**Table 2:** PCR primers for quantitative real-time PCR.

**Primer**	**Forward**	**Reverse**
Bax	5′-TGGAGCTGCAGAGGATGATTG-3′	5′-GCTGCCACACGGAAGAAGAC-3′
Bcl-2	5′-TGGGATGCCTTTGTGGAACT-3′	5′-GAGACAGCCAGGAGAAATCAAAC-3′
BDNF	5′-AAAAAGTTCCACCAGGTGAGAAGA-3′	5′-GCAACCGAAGTATGAAATAACCATAG-3′
RPS29	5′-GCCAGGGTTCTCGCTCTTG-3′	5′-GGCACATGTTCAGCCCGTAT-3′

### Quantification of Mitochondrial Oxidative Damage and Parvalbumin Immunoreactive Interneurons

Coronal frozen sections (40 μm) were used to investigate the anterior cingulate cortex (ACC), a region in the prefrontal cortex known to be sensitive to stress. Triple immunolabeling for labeling DNA oxidative damage, parvalbumin (PV), and perineuronal nets (PNNs) was performed as followed. An antibody against 8-oxo-2′-deoxyguanosine (8-oxo-dG), a product of DNA oxidation, was used as DNA oxidative marker, while the PNNs were labeled with the lectin *Wisteria floribunda Agglutinin* (WFA), which binds the N-acetylgalactosamines in chondroitin sulfate proteoglycans (15, 16). Brain sections containing the anterior ACC were first incubated with PBS + Triton 0.3% +sodium azide (1 g/L) containing 2–3% normal horse serum, then placed for 48 h in a solution with a mouse monoclonal anti-8-oxodG (1:400; AMS Biotechnology, Switzerland) and a rabbit polyclonal anti-parvalbumin (PV) (1:2,500; Swant Inc., Switzerland) primary antibody together with the biotin-conjugated WFA (1:2,000; Sigma, Switzerland). Sections were then washed, incubated with fluorescent secondary antibody conjugates: goat anti-mouse Alexa 488 (1:300; Life Technologies, USA), goat anti-rabbit CY3 (1:300; Chemicon International, USA) and streptavidin 405 conjugate (1:300; Millipore Corporation, USA). Sections were visualized and processed with a Zeiss confocal microscope equipped with ×20 and ×40 Plan-NEOFLUAR objectives. All peripherals were controlled with LSM 710 Quasar software (Carl Zeiss AG, Switzerland). With the triple immunolabeling of 8-oxodG, PV, and WFA, Z-stacks of 12 images with ×20 objective (with a 1.8 μm interval) were scanned (1,024 × 1,024 pixels). Images were filtered with a Gaussian filter to remove background noise and sharpen cell profile contour. Only the inner 9 images of Z-stacks were used for the analyses. The stack images were merged in a tif file with Imaris. Analyses were performed in a delineated region of interest (ROI) comprising the ACC (mainly cg1 and small portion of cg2) by an observer unaware of the experimental groups. To quantify the overall 8-oxo-dG, the staining intensity and number of labeled voxels within the ROI were measured. For PV-IR cell count, PV cell bodies were counted and their mean intensity of labeling (arbitrary unit) also obtained. To analyze the number of PV+ cells surrounded by WFA labeled PNN (WFA+ PV), we used the same “spots module” used to count PV+ cells to assign spot markings on the profile-labeled voxels that fall within a given size. Briefly, the channels for PV and WFA immunolabeling were chosen, and the profile size criterion (>8 and >5 mm, respectively) was defined to quantify stained profiles above these given sizes. The procedure was visually monitored/verified before proceeding. Spots generated for PV that contacted/overlapped with spots generated for WFA (PNN) were considered as those PVI surrounded by PNN (WFA-positive PVI).

### Behavioral Tests

Behavioral tests were performed from P31 to P41. Detailed protocol can be found in (17).$

#### The Elevated Plus Maze (EPM)

The test assesses anxiety-like behavior (17). Rats were placed in the central area, head facing one of the enclosed arms. Number of entries in the open or closed arms, time spent on each arm, head-dipping and rearing were recorded over 5 min to investigate anxiety-like state. The ratio based on the time spent in the closed arms and the time spent in the open spaces (open arms and center of the apparatus) was calculated by time in open space divided by time in closed arms (18).

#### The Open Field (OF)

Motor activity, exploratory drive, and anxiety state were assessed in this test in a circular arena (100 cm diameter) divided in 21 areas. The latency to leave the central circular area, the number of areas crossing and rearing were recorded for 5 min (17).

#### The Novel Object Recognition (NOR)

The novel object recognition (NOR) test was performed in the open field arena during two consecutive days. In the first day, each rat was confronted with two identical objects (A and A′) placed 20 cm apart from the walls of the apparatus. The time exploring each object was recorded for 4 min. In the second day, long-term memory was tested by exposing each animal to two objects in the same open field apparatus: one of the objects used in the first day, considered as the familiar object (A) and a novel (and distinct from A or A′) object (B). Object exploration time for each object was measured and a discriminative index (DI) was calculated by exploration time on object B minus exploration time on object A divided by total exploration time on both objects (19).

#### The Beam Walking (BW)

The beam walking (BW) test was composed of training phase and testing phase. In the first day, rats were trained (three trials) to traverse a wooden beam (width 2 cm, length 100 cm, elevated 70 cm above the floor). They were placed on one side and encouraged to walk to reach a black box on the other side of the beam. In the next day (24 h later) animals were exposed to the same beam and number of hind paw slips were counted as an index of locomotor deficits (17).

#### The Morris Water Maze (MWM)

The Morris water maze (MWM) was done for 6 days (5 days of training and 1 day of test—namely Probe Trial) consecutively in a black tank (200 diameters) filled with water (45 cm depth) at 21 ± 2°C. A black platform (diameter 10 cm) was placed at 2 cm under water level and remained hidden at the same location for the 5 training days. Rats underwent four trials with 10 min inter-trial interval per day and entered the pool facing the wall following a randomized sequence of the four starting points (North, East, South, and West). Latency to find the platform was measured up to 60 s and was considered as a learning indication. If the animal failed in finding the platform within the 60 s, it was conducted to the platform by the experimenter and left during 10–15 s for exploring the room clues. On the 6th day, the probe test trial day, the platform was removed and latency to cross the platform zone and time spent in the platform quadrant were assessed.

### Statistics

For statistical analysis, a one-way ANOVA test followed by Tukey *post hoc* was done for normally distributed data. A Kruskal–Wallis test followed by a Mann–Whitney test (*P* < α/number of tests) was done in case of non-normal distribution. Finally, the mean number of PV+ cells, WFA+PVI, and the overall 8-oxo-dG labeling intensity were compared between the three experimental groups and analyze with ANOVA test followed by a Dunnett test. Significance level was considered when *P* < 0.05 after rectification of *P-*values by Bonferroni correction. All data are presented as mean ± SEM except DTI and NODDI derived parameters as well as PV and WFA cells results expressed as mean ± SD.

## Results

### Caloric Restriction Effects on Maternal and Offspring Weights

**Figure 1:**
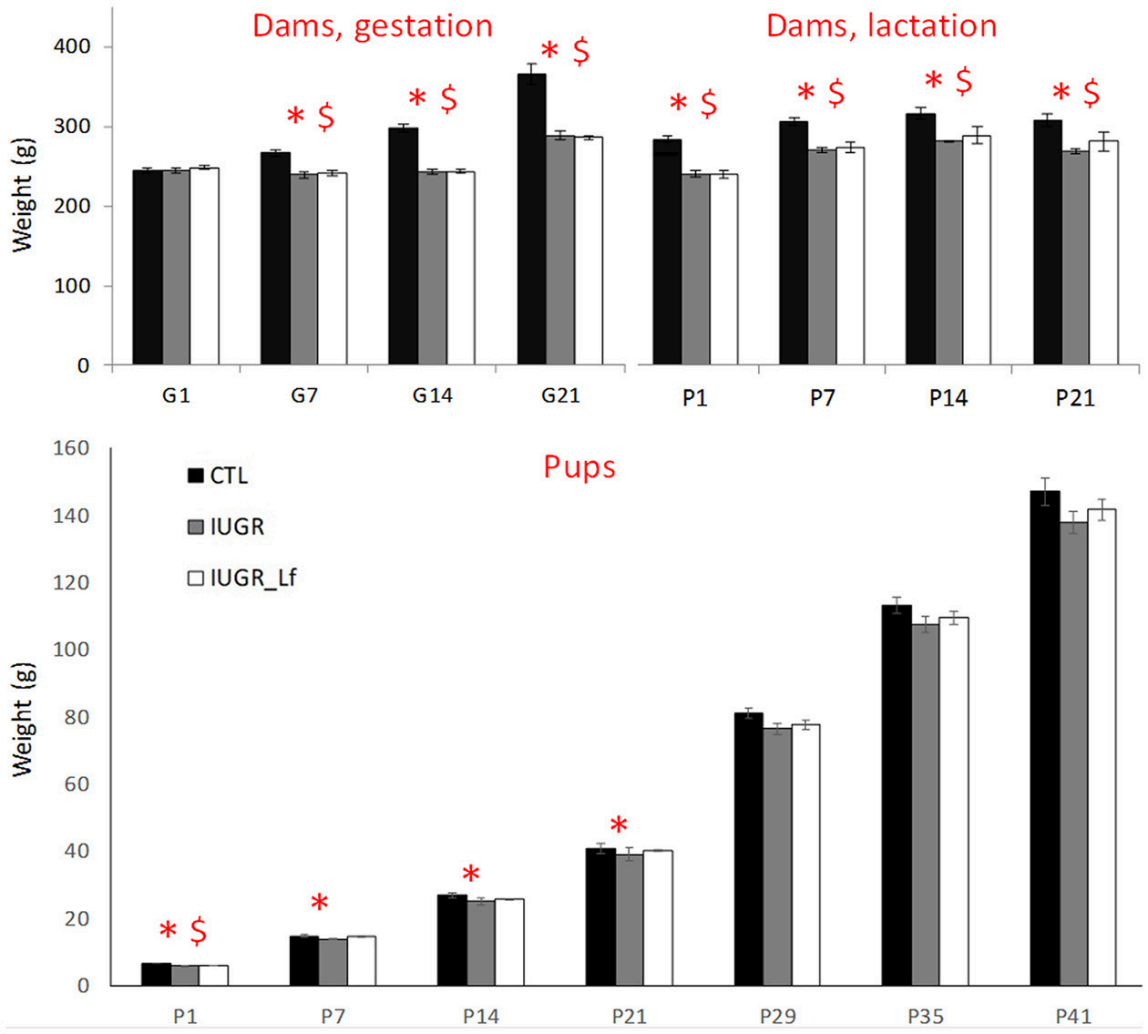
High: dam weight monitoring through gestation (G1 to G21) and lactation until weaning (P1 to P21). Low: rat pups weight monitoring from post-natal day 1 to 41 (P1 to P41). Results are mean ± SEM. *P* < 0.05 *CTL vs. IUGR, $CTL vs. IUGR_Lf.

In order to evaluate the impact of a 50% caloric restriction on dams, weights were measured daily during both gestation and lactation (Figure 1). Maternal body weights were significantly decreased in IUGR and IUGR_Lf groups compared to CTL group. The difference persisted postnatally until weaning. While IUGR and IUGR_Lf gained only 17% of initial weight at gestational day 21 (G21), whereas CTL dams gained 50% of initial weight. No significant effect of Lf supplementation was perceived on the IUGR_Lf dams compared to the IUGR ones. However, IUGR_Lf dam body weights were no longer statistically different from the control groups at the third week of lactation.

At P1 (Figure 1), IUGR and IUGR_Lf pups were lighter than CTL pups. At P7 and 14, only IUGR pup weights were different from CTL group. From P21, pups grew with no significant difference among them.

### Microstructural Integrity

**Figure 2:**
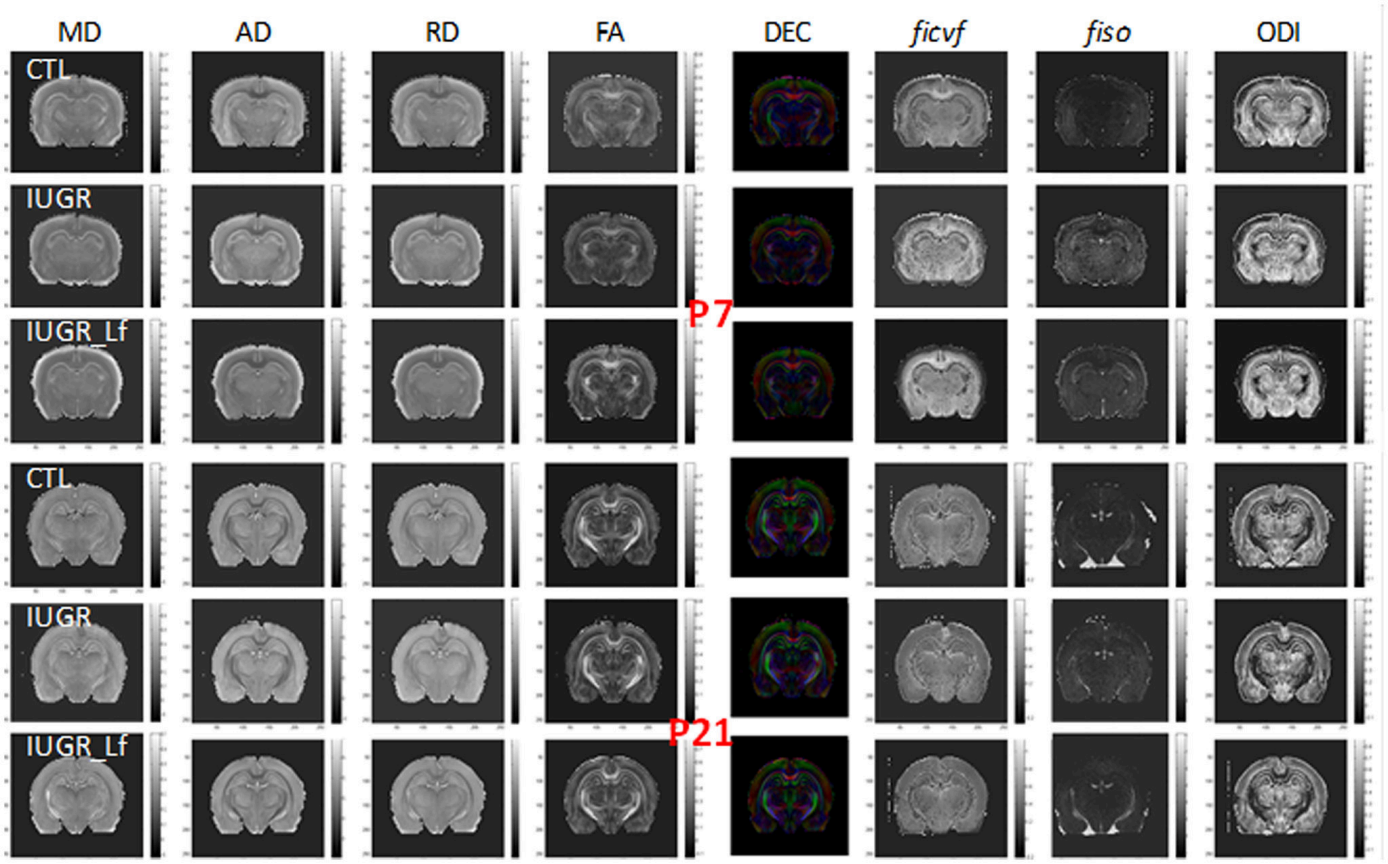
Diffusivity (Mean, MD; Axial, AD; Radial, RD), fractional anisotropy (FA) and direction encoded color (DEC) maps, intra-neurite volume fraction (*f*_*icvf*_), cerebrospinal volume fraction (*f*_*iso*_), and orientation dispersion index (ODI) maps at P7 and P21 for each group.

To allow the assessment of cerebral microstructure, diffusivity (Mean, MD; Axial, AD and Radial, AD), fractional anisotropy (FA) and direction encoded color maps (DEC), intra-neurite volume fraction (*f*_*icvf*_), cerebrospinal volume fraction (*f*_*iso*_) and orientation dispersion index (ODI) maps acquired at P7 and P21 on a 9.4T scanners are presented in Figure 2. High Signal-to-noise ratio and very good resolution were obtained leading to accurate measurement of the DTI and NODDI derived parameters (20).

### Cortical Microstructure

**Figure 3:**
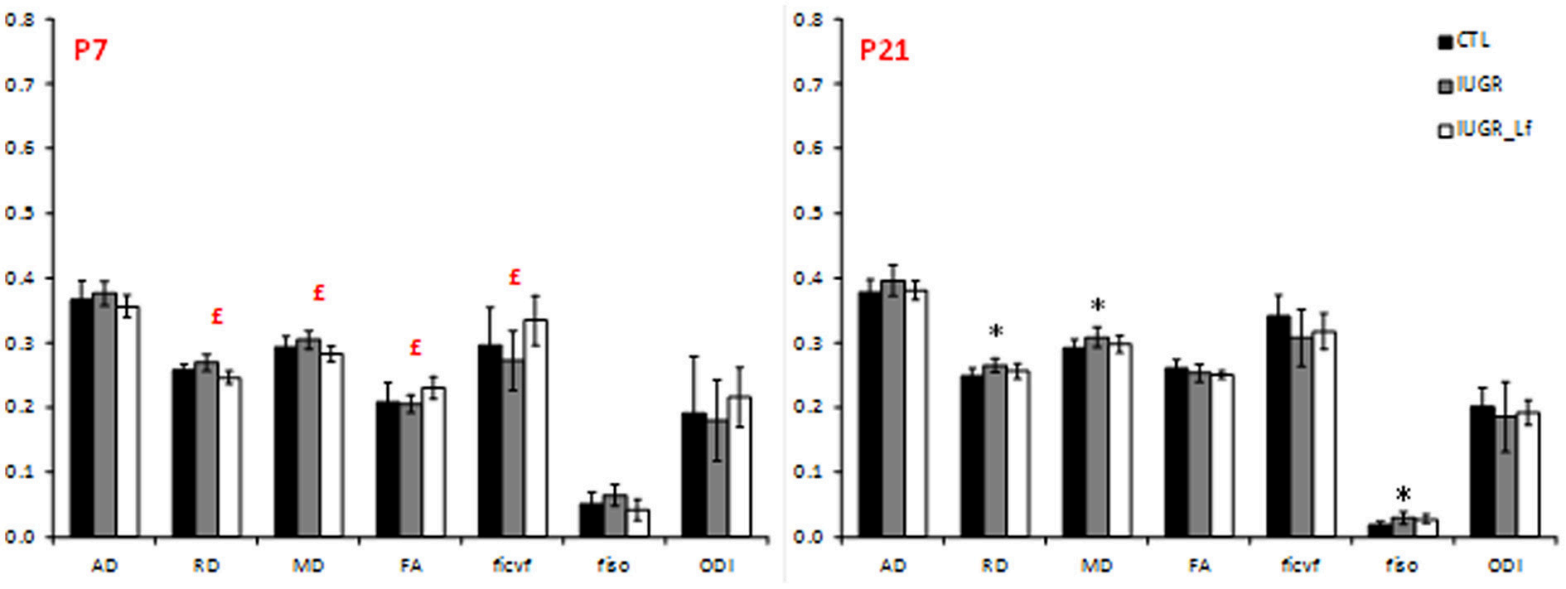
Histogram of diffusivities (Mean, MD; Axial, AD and Radial, RD; × 10^−4^ mm^2^.s^−1^), fractional anisotropy (FA), intra-neurite volume fraction (*f*_*icvf*_), cerebrospinal volume fraction (*f*_*iso*_), and orientation dispersion index (ODI) at P7 and P21 in the cortex. Results are mean ± SD. *P* < 0.05, *CTL vs. IUGR, $CTL vs. IUGR_Lf, £IUGR vs. IUGR_Lf. *Effect of the lesion; £effect of Lf, red positive, black negative; *, £effect of the lesion restored by Lf; *,$effect of lesion but no effect of Lf; *, £,$effect of the lesion partially restored by Lf.

Cortical DTI and NODDI derived parameters assessing cortical microstructure integrity, were obtained by diffusion MR imaging (Figure 3). IUGR and CTL derived parameters were not different from each other at P7, but IUGR_Lf parameters were different compared to the IUGR group: Indeed, a significant decrease in radial (RD) and mean diffusivity (MD), and also increased fractional anisotropy (FA) and intraneurite volume fraction (*f*_*icvf*_) were observed with Lf. At P21, caloric restriction induced significant increase of RD, MD and *f*_*iso*_, this last parameter was not restored by Lf.

**Figure 4:**
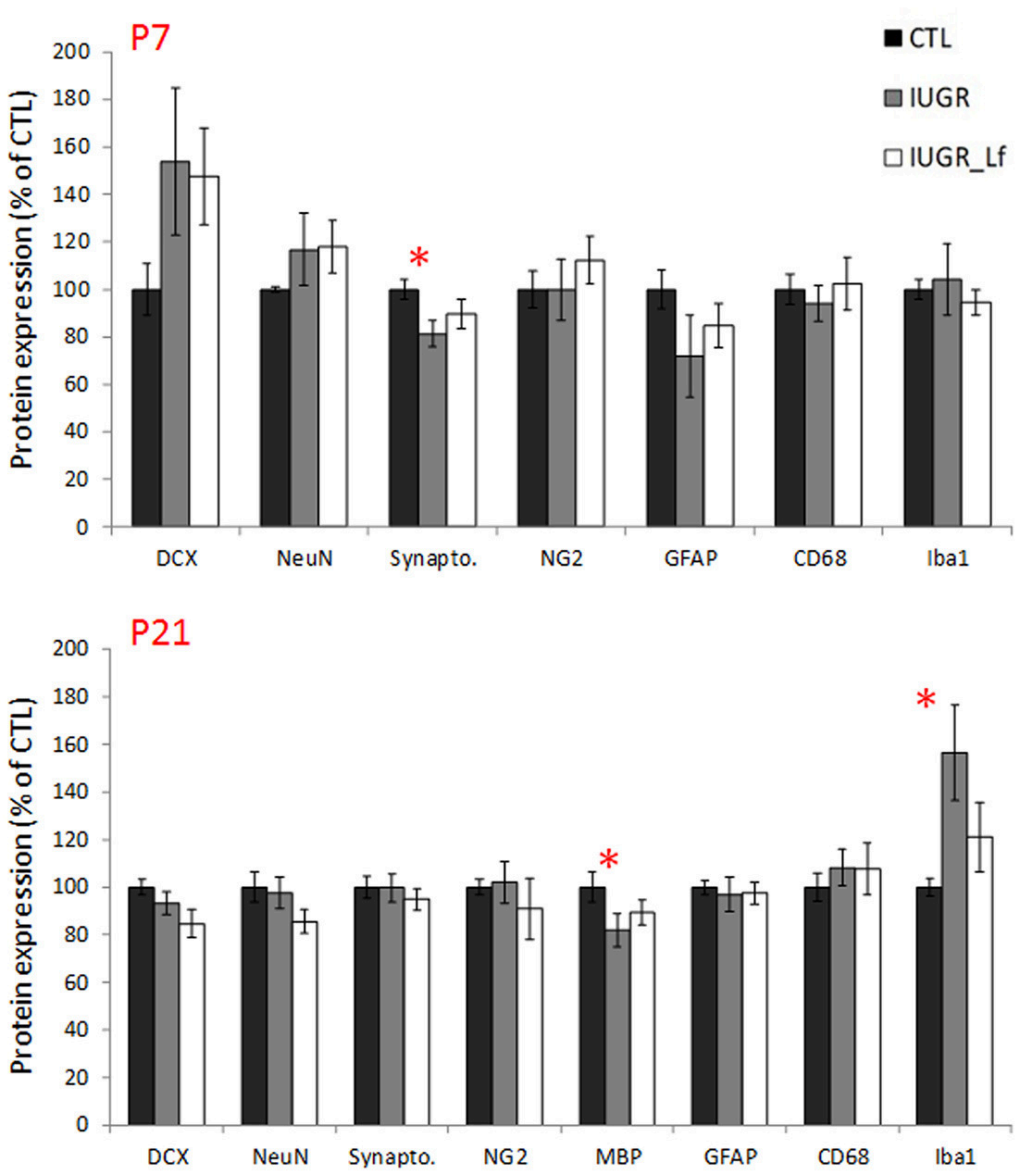
Normalized protein expression to the control group level in CTL, IUGR, and IUGR_Lf pups at P7 (high panel) and P21 (low panel) in the cortex. Results are mean value ± SEM. *N* = 5–7 pups per group (P7) and *N* = 9–14 pups per group (P21). *P* < 0.05 *CTL vs. IUGR. Raw data in Supplementary Tables 1, 2.

In the cortex, most of the cell markers were not modified (Figure 4). At P7, only synaptophysin expression was significantly decreased in the IUGR group compared to CTL, showing a potential delay in synaptogenesis (Figure 4) but expression was similar between IUGR and IUGR_Lf cortices (Figure 4). At P21, only MBP expression was different among the groups (Figure 4). This marker of myelin formation and compaction was significantly decreased in the IUGR group. In the IUGR_Lf group, MBP expression was not statistically different from both IUGR and CTL groups (Figure 4). Also, Iba-1 expression was increased in IUGR pups, suggesting an impact on microglia. However, no alteration in CD68 expression, as marker of reactive microglia, expressions in the IUGR_Lf group were not different from the IUGR group at both ages (Figure 4).

**Figure 5:**
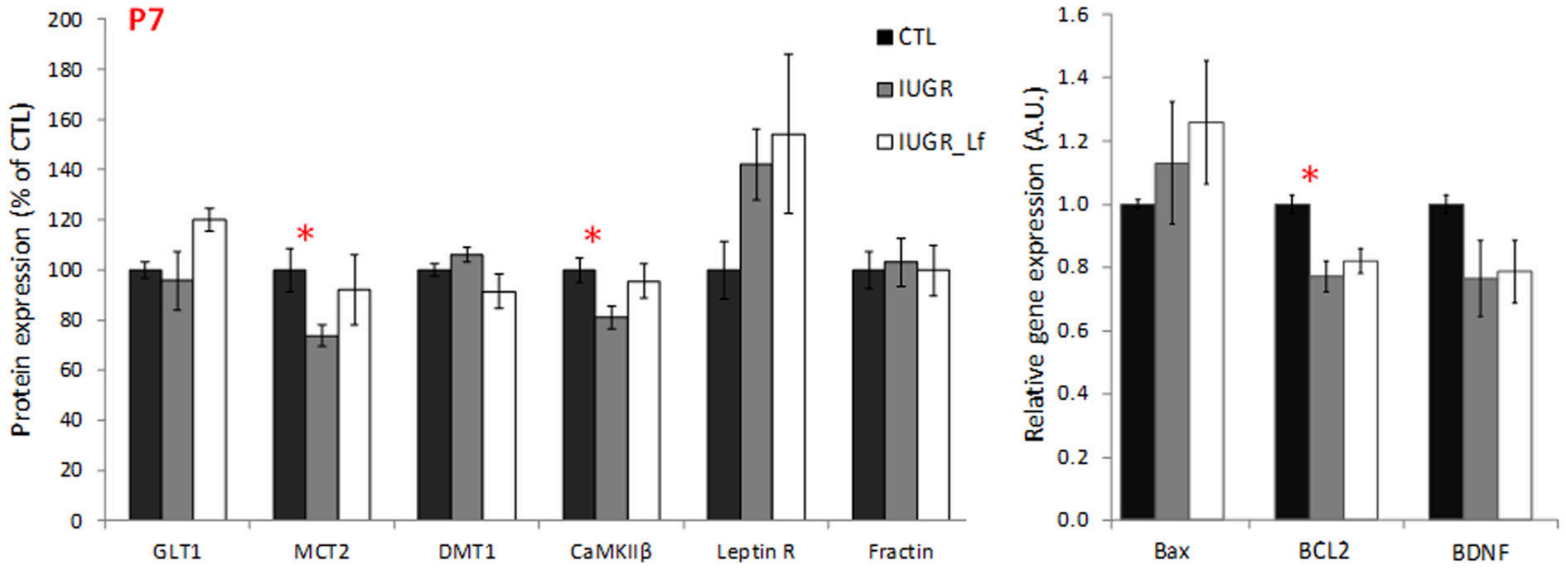
Cortical protein (normalized to the control group level) involved in global brain mechanisms such as metabolism, growth and apoptosis for CTL, IUGR, and IUGR_Lf pups at P7. Quantification was done either after immunoblotting or RT-qPCR. Selection of metabolic transporter and receptor expressions (GLT1, MCT2, DMT1, CaMKIIβ, and Leptin R) and proteic feature of apoptosis (Fractin). Expression of pro- and anti-apoptotic mRNA (Bax and BCL2) and growth factor BDNF mRNA expression (BDNF). Results are mean values ± SEM of *N* = 6–7 pups per group. *P* < 0.05 *CTL vs. IUGR. Raw data in Supplementary Table 3.

Key receptors, transporters and kinases taking part in the global cerebral metabolism were quantified (Figure 5). At P7, MCT2 cortical expression was significantly decreased in the IUGR group (Figure 5), probably as a consequence of disrupted energy metabolism. Also, at P7, a trend was visible with an increased expression of leptin receptor in IUGR pups when compared to CTL pups (*P* = 0.055). βCaMKII, a major kinase in pre- and postsynaptic mechanisms and related to synaptic plasticity, was statistically less expressed in P7 IUGR pups, and showed a trend to be more expressed in IUGR_Lf animals (Figure 5, *P* = 0.086). No statistical difference was observed in the IUGR_Lf group (Figure 5). Protein and gene expressions involved in growth, differentiation and survival, as well as in apoptotic pathways were analyzed by immunoblotting and RT-qPCR. Cortical mRNA levels of BDNF showed a trend (*P* = 0.068) to be decreased (Figure 5) and the Bcl-2 mRNA level was significantly decreased in the P7 IUGR group (Figure 5), whereas no statistical difference with IUGR_Lf was observed (Figure 5) compared to CTL. Fractin expression, known to attest caspase 3 activity, was not statistically different among the groups (Figure 5).

**Figure 6:**
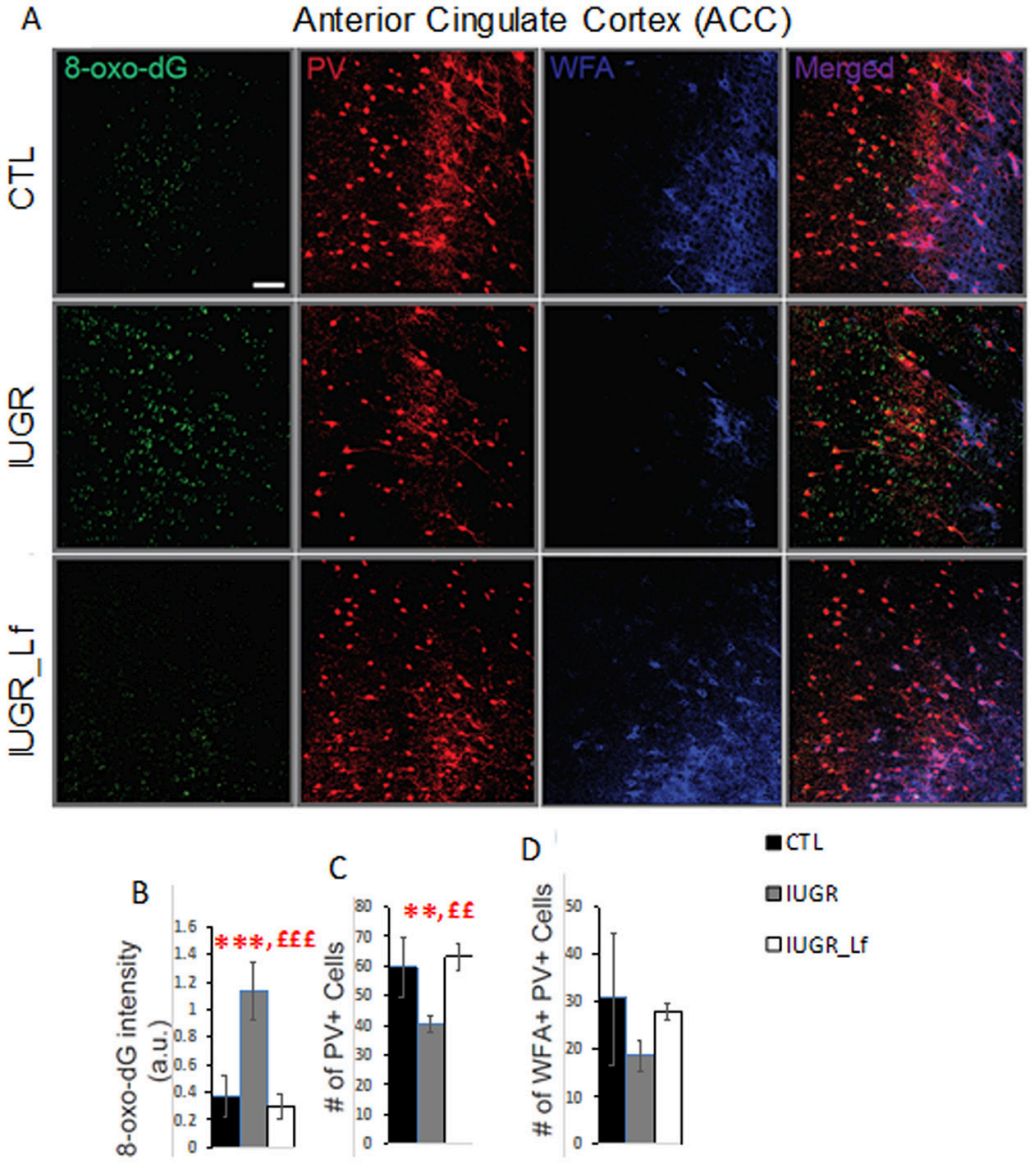
Interneurons assessment in the cortex at P21. **(A)** Micrographs showing labeling for 8-oxo-dG (labels mitochondrial DNA oxidation), PV, and WFA (labels PNN) in ACC of CTL, IUGR, and IUGR_Lf. **(B)** Lactoferrin prevents increase in 8-oxo-dG labeling. **(C)** Quantification reveals that Lactoferrin restores PV immunoreactive cell deficits in hypocaloric induced IUGR rats. **(D)** There is a tendency for lactoferrin to prevent decrease of PNN (WFA + PV) in hypocaloric induced IUGR rats. Bars in all graphs represent SD. For each group *n* = 4 (for each animal 3–4 brain sections). *CTL vs. IUGR, £IUGR vs. IUGR_Lf, number of * or £: 3 *P* < 0.001; 2 *P* < 0.01; Scale bar: 50 μm.

At P21, we assessed whether a hypocaloric induced IUGR would induce oxidative stress in the brain of rat pups, leading to impairment of the cortical parvalbumin (PV) expressing neurons, known to play a critical role in cognition. A significantly higher 8-oxo-dG [marker for DNA oxidative damage (21)] in the cortex of hypocaloric induced IUGR pup rats compared to control was observed (*P* < 0.0001, Figure 6). We have previously established that oxidative stress during development leads to PV-IR and WFA+ PV cell deficit (22, 23). Indeed the number of PV-IR cell and PV-IR cell intensity in the ACC of IUGR rats were significantly decreased compared to CTL (Figure 6, # of PV cell: *P* = 0.0044, and PV-IR cell intensity: *P* = 0.047, not shown). However, the WFA+ PV cells, which are PV+ cells surrounded by PNN were not strongly affected as the decrease in IUGR rats *vs*. CTL remained at a trend level.

**Figure 7:**
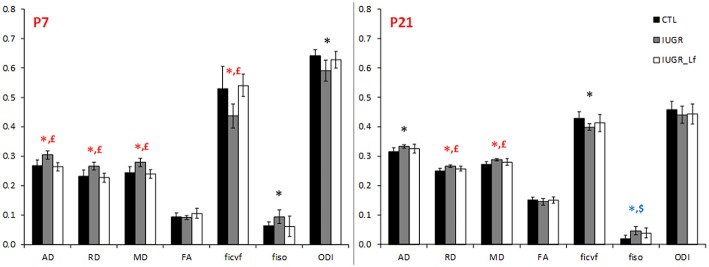
Histogram of diffusivities (Mean, MD; Axial, AD and Radial, RD; × 10^−4^ mm^2^.s^−1^), fractional anisotropy (FA), intra-neurite volume fraction (*f*_*icvf*_), cerebrospinal volume fraction (*f*_*iso*_), and orientation dispersion index (ODI) at P7 and P21 in the striatum. *P* < 0.05, *CTL vs. IUGR, $CTL vs. IUGR_Lf, £IUGR vs. IUGR_Lf. *Effect of the lesion; £effect of Lf, red positive, black negative; *, £effect of the lesion restored by Lf; *,$effect of lesion but no effect of Lf; *, £,$effect of the lesion partially restored by Lf.

In order to assess the subsequent damages of oxidative stress on PV-IR cells and WFA+ PV (PNNs) during early post-weaning ages, we assessed whether Lf could prevent PV-IR and PNN impairment in ACC of IUGR rats. Indeed, Lf supplementation during development prevented increase of 8-oxo-dG intensity in the ACC of IUGR_Lf rats as compared to IUGR rats (*P* < 0.0001, Figure 7). The oxidative stress marker levels, 8-oxo-dG intensity, in ACC of IUGR_Lf rats did not significantly differ from that of in control rats. Lactoferrin also fully restored the number of PV-IR cells in ACC of IUGR_Lf compared to IUGR alone (*P* = 0.001), and compared to CTL rats (*P* = 0.65, NS). Interestingly, the WFA+ PV (PV PNNs) deficit in ACC of IUGR rats was not prevented by Lf (*P* = 0.23, NS).

### Striatum

DTI and NODDI derived parameters were measured in the striatum (Figure 7). IUGR striatal microstructure integrity presented significant alterations compared to the CTL group. Increase in AD and RD at P7 and P21, as well as decrease in *f*_*icvf*_ and increase in *f*_*iso*_ at P7 depicted microstructural changes. DTI and NODDI parameter changes were reverted in P7 IUGR_Lf pups and partially at P21.

**Figure 8:**
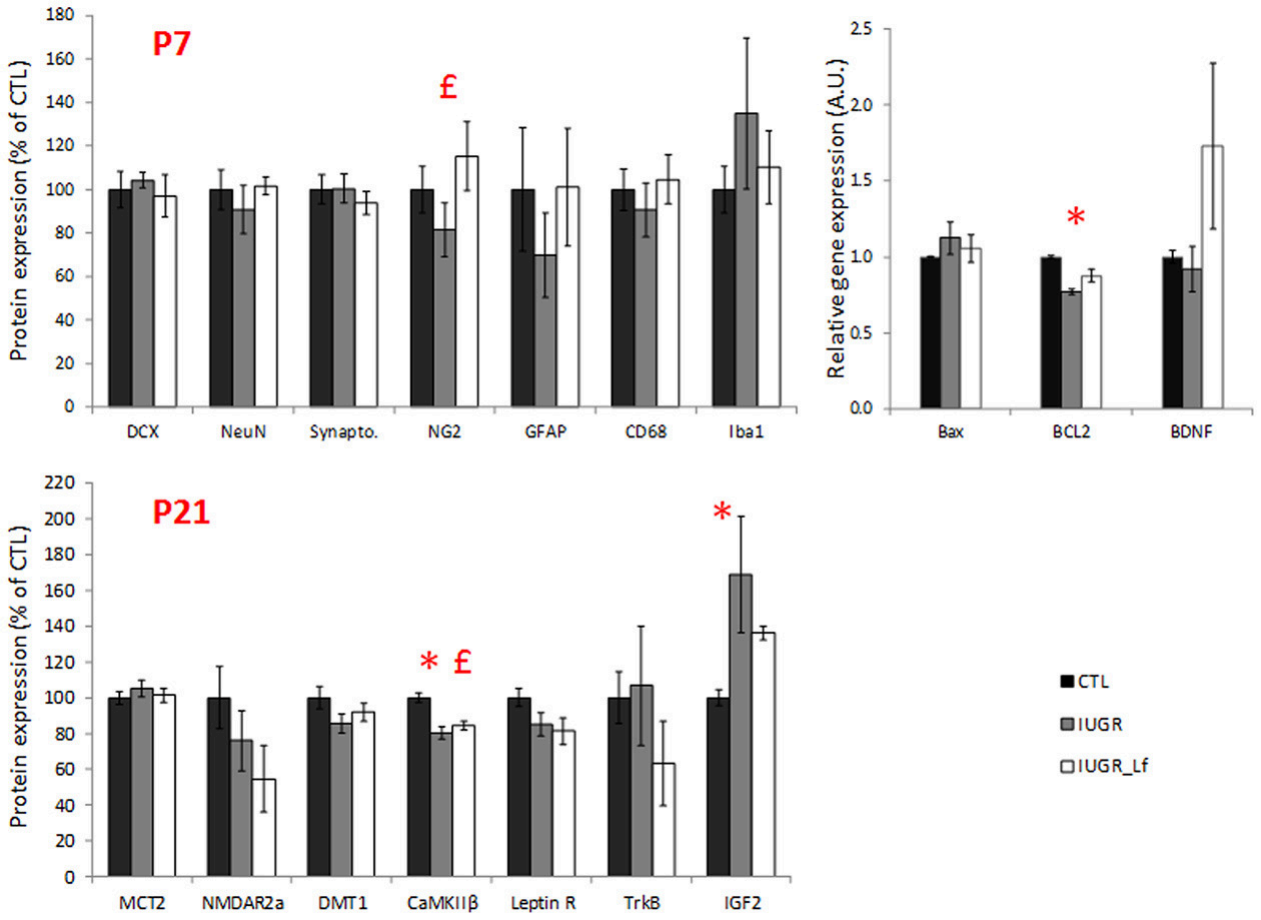
Striatal protein (normalized to the control group level) for CTL, IUGR, and IUGR_Lf pups at P7 (high panel) and P21 (low panel). Structural protein expression at P7 (DCX, NeuN, Synapto., NG2, GFAP, CD68, and Iba1). Expression of striatal pro- and anti-apoptotic mRNA at P7 (Bax and BCL2). Selection of metabolic transporter and receptor expressions in the striatum at P21 (MCT2, NMDar2a, DMT1, CaMKIIβ, and Leptin R). Growth factor molecule and receptor mRNA expression in the striatum at P21 (TrKB and IGF2). Results are mean values ± SEM of *N* = 3 to 14 pups per group. *P* < 0.05 *CTL vs. IUGR, £IUGR vs. IUGR_Lf. Raw data in Supplementary Tables 4, 5.

Structural proteins used as cell markers in the striatum were not differentially expressed between the groups. However, NG2 expression was increased at P7 in IUGR_Lf striatum compared to the IUGR group (Figure 8). In striatum, at P7, the apoptotic pathway was potentially affected in IUGR pups with the anti-apoptotic Bcl-2 mRNA expression statistically downregulated and data showed a tendency (*P* = 0.053) to be reversed in IUGR_Lf when compared to IUGR pups. At P21 (Figure 8, lower panel), alteration in synaptic plasticity in the IUGR group was depicted by a statistical decrease in βCaMKII expression compared to the CTL group. This altered expression was reverted in the IUGR_Lf group. Fractin quantification was not statistically different among the groups. Growth factor IGF2 was found to be statistically increased in IUGR at P21 with no statistical difference in IUGR_Lf.

### External Capsule

**Figure 9:**
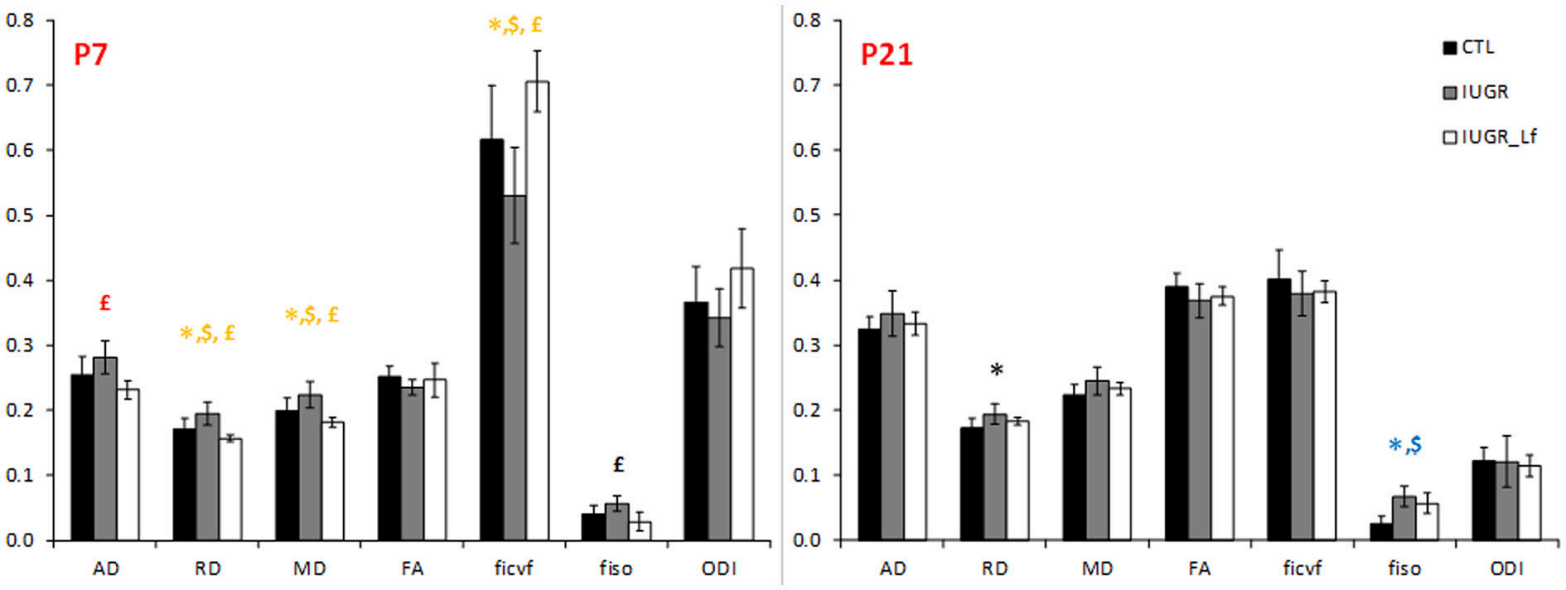
Histogram of diffusivities (Mean, MD; Axial, AD and Radial, RD; × 10^−4^ mm^2^.s^−1^), fractional anisotropy (FA), intra-neurite volume fraction (*f*_*icvf*_), cerebrospinal volume fraction (*f*_*iso*_), and orientation dispersion index (ODI) at P7 and P21 in the external capsule. *P* < 0.05, *CTL vs. IUGR, $CTL vs. IUGR_Lf, £IUGR vs. IUGR_Lf. *Effect of the lesion; £effect of Lf, red positive, black negative; *, £effect of the lesion restored by Lf; *,$effect of lesion but no effect of Lf; *, £,$: effect of the lesion partially restored by Lf.

DTI and NODDI derived parameters in external capsule white matter tract are shown in Figure 9. At P7, RD was significantly increased in IUGR pups and reversed, along with a decrease in AD and MD, in IUGR_Lf when compared to IUGR. In the P7 IUGR_Lf group, *f*_*icvf*_ and *f*_*iso*_ were also significantly different from the IUGR group and there was no statistical difference between IUGR_Lf and CTL pups.

### Behavioral Tests

Cognitive and motor functions were assessed by a battery of behavioral tests performed between P31 to P41. These tests target behavioral aspects that could be impaired in IUGR children, such as motor activity, attention, memory and anxiety. Rats were frequently observed and monitored; pups from IUGR groups did not exhibit any abnormalities in motion, activity or basic behavior compared to the controls.

### Elevated Plus Maze (EPM)

**Figure 10:**
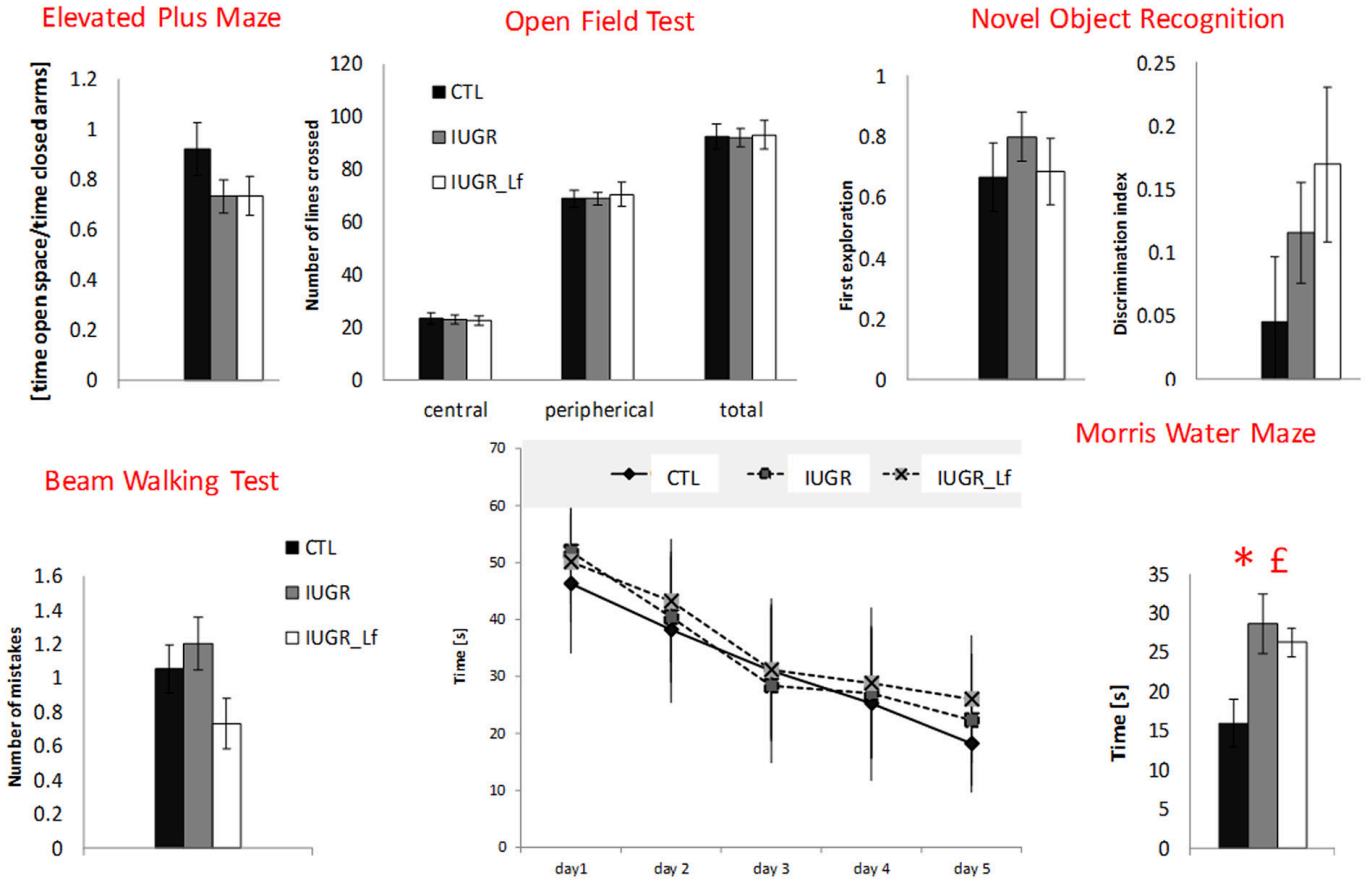
Behavioral test results for CTL, IUGR, and IUGR_Lf pups. Elevated plus maze: ratio measuring the time spent in open space divided by the time spent in closed arm in the elevated plus maze. The center of the apparatus was considered as open space. Open field test: exploratory drive analysis by number of lines crossed in the central and peripherical zone, and in total in the open filed test. Novel object recognition: preferences between familiar and new object in novel object recognition. First object explored (familiar object = 0 and new object = 1) and Discrimination index showing preference for the new object. Beam walking test: number of paw slips during the beam walking test. Morris water maze: time to find the platform zone in the Morris water maze with learning curve displaying the time to find the platform through the five-day training (Day values are the mean of four trials) and time to find the platform zone the day of the test. All results are mean values ± SEM, *N* = 16–26 pups per group. *P* < 0.05 *CTL vs. IUGR, £IUGR vs. IUGR_Lf.

The elevated plus maze assesses the motor activity as well as the anxiety level. The global activity between the groups was not different and showed the three groups staying more in the closed arms than in the open ones (Figure 10). Ratio of time spent in the open to time spent in the closed arms did not show any difference among groups.

### Open Field Test (OFT)

This test assesses the exploratory drive, as well as the global activity. Rats usually avoid open spaces and rather prefer exploring the area close to the wall (thigmotaxis). This trend was visible within the three groups (Figure 10). However, no differences were observed.

### Beam Walking Test (BWT)

Locomotor activity and coordination were assessed by BWT. The number of paw slips was converted into digits and counted as number of mistakes. No statistical differences were found between the groups (Figure 10).

### Novel Object Recognition (NOR)

The NOR test was used to assess long-term memory. Rats were confronted to two objects in the 2 sessions of testing: two familiar objects (*objects A and A*′*)* and a new one (*object B)*. The data did not detect preferences for the new objects nor differences within groups (Figure 10).

### Morris Water Maze (MWM)

In the day of the test (Probe Trial) when the platform was removed, the time to find the platform zone was measured (Figure 10). CTL pups performed significantly better, crossing the platform region faster than IUGR and IUGR_Lf groups. The ability to learn did not vary between the groups during the training phase.

## Discussion

In the present study, we explored the potential neuroprotective action of maternal bovine lactoferrin (Lf) supplementation on a 50% CR-induced IUGR model. As IUGR is thought to induce a delay in neurodevelopment, we addressed special focus on brain microstructural integrity, cell markers as well as metabolic proteins and gene expressions, which are important in cerebral development and injury. Further investigations were pursued for behavioral deficits. Analyses were performed at P7, P21 and from P31 to P41. P7 corresponds to the near term equivalent age for humans. P21 corresponds to the peak of myelination and cortical maturation providing already good information about the consequences of IUGR and potential Lf neuroprotection. Behavioral tests were performed at a time period where myelination is nearly completed (P31 to 41, preadolescent human equivalent) and as such potential mental disabilities can be observed in children at this time point. Indeed, these tests target behavioral aspects that could be impaired in IUGR children, such as motor activity, attention, memory and anxiety.

### IUGR Model

Despite any effect of Lf supplementation on the weight of the dams, evidences validated our CR-induced IUGR model as it reduced maternal weight gain during gestation and had long-lasting effect during lactation period. While control dams gained 50% of their initial body weight by the end of gestation, restricted animals gained only 17%. This reduced maternal weight gain was also observed in a previous study using a stress induced model (maternal dexamethasone infusion) during the third week of gestation (12) and by others (24). Low birth weight in IUGR newborns is considered as a major indicator of prenatal insults. Pups were weighted at P1, and as both restricted diets were isocaloric, it was not expected that nutritional supplementation with Lf would improve low body weight in P1 IUGR pups indicating that offspring birthweight is highly dependent on the maternal food caloric intake during gestation. However, while IUGR pups receiving Lf through lactation caught up control body weight at P7, IUGR catch-up was only observed at P21. This gives rise to a potentially improved early metabolism in IUGR pups receiving Lf. In a placental insufficiency model, pups with moderate IUGR had a catch-up growth at P7, whereas catch-up growth in severe IUGR pups was not reported (25).

### Cortical Impairment and Effects of Lactoferrin

We reported a decrease in cortical synaptophysin expression at P7 in whole cortical homogenate tissue, resulting from caloric restriction. As IUGR neurodevelopment is thought to be delayed due to reduced nutrients during a critical phase of cerebral development (26–28), the present result suggest that IUGR leads to transient delay in synaptogenesis in the cortex in P7 restricted rats. Similarly in sheep (29), impaired fetal environment was induced by an intra-amniotic lipopolysaccharide pro-inflammatory environment and cerebral synaptophysin expression was reported to be decreased in motor, somatosensory and entorhinal cortices causing reduction in density of presynaptical boutons.

A decrease in beta Ca2+/calmodulin-dependent protein kinase II (βCaMKII) expression in the cortex further supports this interpretation. βCaMKII is activated by calcium and is an important kinase targeting substrate responsible for long-term potentiation (30). *In vitro* overexpression induced an increase in the number of neurite extensions and in formation of new synapses (31). Therefore, it is related to morphology and quantity of synapses (32) by modifying the cytoskeletal structure. Along with our previous results on synaptophysin expression, decrease in βCaMKII emphasizes a potentially altered number/size of synapses and supports a transient delay in synaptic formation and possibly function.

Myelin basic protein (MBP) is one of the main components of myelin sheath structure and primordial for its formation and compaction and for axonal. In the current study, MBP expression was reduced in the cortex in IUGR pups at P21. Uterine artery ligation IUGR induced models have been widely used to approach white matter damage linked to perinatal impairments such as prematurity or fetal growth restriction observing a similar reduction in MBP density in the corpus callosum and the cingulum, sign of myelination defect (24).

Moreover, cortical expression of Iba-1, a microglial protein, was increased at P21. As it is expressed in both quiescent and amoeboid form and even though it is upregulated by activation (33), we quantified CD68 expression in order to identify properly activated microglial. However, CD68 analysis failed to identify microglial activation induced by IUGR. In a placental insufficiency rat model, inflammatory microgliosis was reported to impair white matter development (24). Indeed, using another marker (OX42) for immunohistochemistry, a higher density of microglia was observed in IUGR rats. Taken together, myelin integrity alteration concomitant with increased microglia density are in accordance with others work (14). Even though, our current results showed comparatively less microstructural alterations, it may further demonstrate the neuroinflammatory basis for cerebral impaired development in IUGR condition.

Despite an absence of significant differences, a tendency in the up-regulation of βCaMKII, synaptogenesis and MBP levels was observed in the Lf supplemented group providing evidence for a beneficial effect on cortical development. Indeed, DTI and NODDI derived parameters demonstrated a potential conservation of the microstructure in the cortex at P7 in IUGR_Lf rats compared to IUGR even though at P21 remaining impairments detected by diffusion MRI appeared moderates in the IUGR group. Altogether these results suggest that Lactoferrin reduces the delayed and impaired cortical development following caloric restriction.

We have demonstrated that redox dysregulation and oxidative stress (genetic or environmental origin) during development, in several animals models relevant to schizophrenia and autism, at microcircuit levels, leads to excitatory-inhibitory imbalance through impairment of the PV inhibitory network and their PNN (23, 34, 35). Similar redox dysregulation processes during development, at macrocircuit levels, could underlie impaired oligodendrocytes and delayed cortical myelination (34, 36). Parvalbumin immunoreactive (PV-IR) cell, oligodendrocyte and myelination impairment have been reported in schizophrenia (37). Abnormal control of redox system could therefore affect neuronal synchronization important for cognitive processing, and also disturb myelination leading to subsequent white matter injuries. N-acetyl-cysteine (NAC) is an antioxidant demonstrated to prevent oxidative stress during development and as such, to protect PVI and PNN. Clinically, several positive effects of NAC have been observed including an improvement of negative symptoms, a decrease of antipsychotics side effects, and an improvement of mismatch negativity and local neural synchronization in chronic schizophrenic psychosis. Recently, Conus et al. (38) observed on early psychosis patients that NAC add-on therapy for 6 months led to significant improvements in neuro-cognition and a reduced positive symptoms in patients presenting high oxidative status.

In the present study, lactoferrin prevents oxidative stress thus restoring PV-IR cell deficits in ACC of hypocaloric induced IUGR rat pups. Lactoferrin could have prevented oxidative stress and restored PV-IR impairment through anti-inflammatory and antioxidant properties in the same way as NAC does, even without improving MBP expression (myelination). Although beyond the scope of the present study, it may prove vital for the future studies to look into additional functional consequences of hypocaloric induced IUGR, such as mismatch negativity (sensory gating), and fast spiking neuronal synchronization (important for cognitive and social processing), and the effect of lactoferrin supplementation. This would certainly shed additional light on the contribution of a developmental undernutrition in psychiatric disease such as schizophrenia and potential prevention.

### Impaired Microstructure in Striatum and Restoration With Lf

MR Imaging of the striatum of IUGR pups showed promising improvement of the neurodevelopment with Lf. The striatum is a vulnerable gray matter area in developing brain (11), which was proposed to be especially affected in case of periventricular leukomalacia and may be related to mild motor deficits (39) but also potentially to psychiatric disorders such as schizophrenia (40). In the present study, at P7, microstructural organization was altered or delayed, as depicted by reduction of the neurite density (*f*_*icvf*_) and dispersion of the fibers (ODI) were reduced whereas isotropic volume fraction (*f*_*iso*_) was increased. No changes in cell markers used in the current study were identified, suggesting alterations of arborization/maturation rather than in neuronal or glial reduced populations. No difference in MBP expression was observed, however, the low level of MBP immunostaining of brain sections at early time-point did not allow a structural assessment in the axonal fascicles. Nonetheless, these parameters were normalized with Lf, promoting a better integrity of the striatum in the first week after birth. Similar neuroprotective effect of Lf was observed at longer term (P21) in the striatum following lipopolysaccharide injection in the rat pup (11). MCT 2 expression in the striatum was decreased as observed in the cortex. Similar to the cortex, striatal mRNA expression of Bcl-2 was also downregulated and normalized with the Lf supplementation. As Bax expression was not affected by IUGR, reduction in anti-apoptotic protein presumed an increased apoptotic rate in IUGR condition (Bax/Bcl-2) (41). However, no increase in fractin expression was visible. Interestingly, βCaMKII protein expression was reduced in IUGR and restored with Lf at P21. Lactoferrin administration also improved striatal expression of βCaMKII. DTI/NODDI and βCaMKII findings suggest reduced arborization as consequence of IUGR reversed by Lf.

### External Capsule, IUGR Impact on White Matter Tracts, and Repair With Lf

In external capsule, increased water diffusivity was observed perpendicularly to the main diffusion direction at P7. This abnormal RD was associated with increased *f*_*iso*_ and reduced neurite density emphasizing clearly for a defective myelin organization. RD in the external capsule and corpus callosum were also increased in P21 moderate—but not in mild—IUGR pups after placental insufficiency (42). As it was correlated with poor oligodendrocyte density and an abnormal percentage of unmyelinated axons, RD modification was suggested to result readily from this myelin injury. All these studies related impaired neurodevelopment with alteration in the external capsule around 3 weeks of age. By this time, oligodendrocyte and astrocyte proliferations and maturations stage is likely to be finished. However, in our case, the microstructural alterations of external capsule were observed in P7 pups transiently as almost no difference was measured at P21. Positive effect of Lf at 7 days point was obvious. Our model further argues for a potential delay in myelination process rather than a long-lasting injury.

Neuroprotection with Lf was assessed in the inflammatory (11) and hypoxic-ischemic (10) models, as well as in the present CR model. While partial normalization was observed in the case of brain inflammation, diffusivities were fully normalized in the hypoxic-ischemic injured brain, such as the RD in the present study. Supplementation with Lf appeared to improve impaired white matter formation.

### Limitation of IUGR Impact and Neuroprotection by Lf

The present study further illustrates the complexity of the IUGR condition, which depends on the timing, the severity, the heterogeneity of risk factors and pathways potentially impaired that are mimicked by various animal models. Maternal stress, placental insufficiency and undernutrition mimicking-models were all reported to interfere with fetal trajectory growth but altered pathways are very likely to differ depending on the risk factor (43). Our present model, moderate undernutrition, using a 50% caloric restriction during gestation, is likely to induce a mild and transient impairment to the neurodevelopment despite the IUGR phenotype. We believe that part of the non-significant changes seen in the model and the partial Lf neuroprotection are due to the mild IUGR model used. It is important to notice that mild IUGR has been also reported in humans with moderate but depicted developmental impairments (44). It is important to notice that this model mimics one type of environmental risk which is necessary but not sufficient by itself alone to reproduce fully the clinical phenotypes. One may need the combination with genetic risks as well. As such, the mildness of the model is evident and may be responsible for absence of behavioral impairments. Furthermore, behavior evaluation was made at childhood age before adulthood where psychiatric symptoms generally occur following IUGR including social problems, poor cognitive performances, anxiety, and schizophrenia (45). Notice that, due to the variability of the caloric restriction model and according to the high number of different techniques used in this study, number of pups in each group was not large enough to assess possible gender differences but it is point to consider in further experiments.

## Conclusion

In this study, we show that IUGR induced by moderate undernutrition demonstrated mild cerebral impairments. Cortical synaptogenesis and myelination were potentially delayed. Striatal microstructure showed alterations. The white matter integrity assessed in the external capsule presented abnormal development. Lf supplementation showed beneficial restoration in the cortex, in the striatum microstructure and in the white matter organization of the external capsule. Further Lf showed reduced oxidative stress in ACA interneurons. This model does not fully represent the clinical situation as IUGR newborns often cumulate adverse conditions in addition to IUGR, such as hypoxia-ischemia or infection/inflammation. Nevertheless, oral Lf supplementation showed partial neuroprotection in this CR IUGR model. This is also neuroprotective against hypoxia-ischemia and inflammation in the developing brain, and thus represents a very promising tool to reduce developmental diseases in IUGR and preterm infants.

## Author Contributions

All authors participated to the design of the study. ES performed behavioral analysis. AT performed IUGR model. CL and J-HC performed immunostaining, protein and gene expression analysis. YvdL performed MRI experiments and analyzed the data. YvdL and CL wrote the manuscript. ES, J-HC, AT, KD, and SS revised the manuscript.

### Conflict of Interest Statement

The authors declare that the research was conducted in the absence of any commercial or financial relationships that could be construed as a potential conflict of interest. The handling Editor declared a past collaboration with several of the authors, YvdL and SS.
