# Supplementary material for: Nutritional Intervention for Developmental Brain Damage: Effects of Lactoferrin Supplementation in Hypocaloric Induced Intrauterine Growth Restriction Rat Pups
Source: Front Endocrinol (Lausanne). 2019 Feb 8;10:46. doi: 10.3389/fendo.2019.00046 (PMC6375847; doi:10.3389/fendo.2019.00046)
Supplement: Supplementary file 1 [file Table_1.docx]

| **P7 Cortex** | **DCX** | **NeuN** | **Synapto** | **NG2** | **GFAP** | **CD68** | **Iba1** |
| --- | --- | --- | --- | --- | --- | --- | --- |
| **CT_1** | 77.4 | 101.1 | 112.0 | 86.5 | 113.3 | 81.6 | 92.2 |
| **CT_2** | 138.4 | 98.9 | 96.3 | 98.2 | 101.1 | 110.0 | 107.8 |
| **CT_3** | 84.2 | 100.0 | 91.7 | 115.3 | 85.6 | 108.4 |  |
| **CT_4** | 94.2 | 94.8 | 108.3 | 128.8 | 106.3 | 87.1 | 117.7 |
| **CT_5** | 128.8 | 101.2 | 86.3 | 72.9 | 76.5 | 89.7 | 90.1 |
| **CT_6** | 77.1 | 104.0 | 105.5 | 117.0 | 138.1 | 123.1 | 97.0 |
| **CT_7** |  |  |  | 81.3 | 79.1 |  | 95.2 |
| **IUGR_1** | 83.0 | 136.4 | 81.4 | 100.7 | 95.5 | 110.9 | 95.4 |
| **IUGR_2** | 76.2 | 80.1 | 63.5 | 81.3 | 44.5 | 58.6 | 79.5 |
| **IUGR_3** | 130.1 | 61.6 | 71.3 | 107.8 | 20.2 | 101.7 |  |
| **IUGR_4** | 205.4 | 137.9 | 102.9 | 153.5 | 113.9 | 101.5 | 69.4 |
| **IUGR_5** | 274.0 | 157.0 | 83.9 | 97.4 | 85.3 | 100.4 | 149.2 |
| **IUGR_6** | 154.4 | 127.7 | 85.3 | 58.3 |  | 91.8 | 128.7 |
| **IUGR_Lf_1** | 128.2 | 113.0 | 84.8 | 136.3 | 72.1 | 115.4 | 79.6 |
| **IUGR_Lf_2** | 84.8 | 155.9 | 74.8 | 142.3 | 106.4 | 161.8 | 96.3 |
| **IUGR_Lf_3** | 80.0 | 96.8 | 67.0 | 134.5 | 74.8 | 93.5 | 99.4 |
| **IUGR_Lf_4** | 224.7 | 112.7 | 103.5 | 102.3 | 73.3 | 91.0 | 81.5 |
| **IUGR_Lf_5** | 194.2 | 83.9 | 87.7 | 104.0 | 127.2 | 74.9 | 99.1 |
| **IUGR_Lf_6** | 169.3 | 160.6 | 114.1 | 69.5 | 52.8 | 94.3 | 86.0 |
| **IUGR_Lf_7** | 152.5 | 102.3 | 96.2 | 97.5 | 87.1 | 86.2 | 120.1 |

Table 1: Protein optical density normalized to actin (42 kDa) quantified for each rat (from CTL, IUGR and IUGR_Lf pups) at P7 in the cortex corresponding to figure 4, high panel. Proteins were quantified using CTL group intensity average as 100%. (n=5-7 animals per group).

| **P21 Cortex** | **DCX** | **NeuN** | **Synapto.** | **NG2** | **MBP** | **GFAP** | **CD68** | **Iba1** |
| --- | --- | --- | --- | --- | --- | --- | --- | --- |
| **CT_1** | 100.1 | 112.2 | 113.6 | 94.3 | 97.4 | 91.9 | 134.7 | 114.4 |
| **CT_2** | 108.3 | 122.4 | 115.0 | 108.3 | 101.4 | 108.7 | 69.8 | 100.7 |
| **CT_3** | 91.5 | 65.4 | 71.4 | 97.4 | 101.2 | 99.3 | 95.5 | 84.9 |
| **CT_4** | 116.6 | 127.6 | 114.3 | 98.5 | 66.1 |  | 99.8 | 110.0 |
| **CT_5** | 102.4 | 78.7 | 93.7 | 84.5 | 95.6 | 111.5 | 102.5 | 91.5 |
| **CT_6** | 80.9 | 93.7 | 91.9 | 117.1 | 138.3 | 88.5 | 97.7 | 98.5 |
| **CT_7** | 107.2 | 93.0 | 109.5 | 113.9 | 123.5 | 90.8 | 83.7 | 101.9 |
| **CT_8** | 92.8 | 107.0 | 90.5 | 86.1 | 76.5 | 109.2 | 116.3 | 98.1 |
| **CT_9** | 97.7 | 87.9 | 96.0 | 103.4 | 97.7 | 92.9 | 84.2 | 83.7 |
| **CT_10** | 102.3 | 112.1 | 104.0 | 96.6 | 102.3 | 107.1 | 115.8 | 116.3 |
| **IUGR_1** | 101.4 | 81.9 | 67.1 | 135.0 | 96.4 | 70.6 | 107.2 | 122.6 |
| **IUGR_2** | 79.6 | 52.3 | 58.4 | 162.1 | 55.9 | 120.6 | 66.8 | 117.0 |
| **IUGR_3** | 136.4 | 127.5 | 94.1 | 101.5 | 99.0 | 64.6 | 134.1 | 189.0 |
| **IUGR_4** | 122.2 | 112.3 | 98.0 | 76.0 | 107.0 | 68.7 | 118.3 | 93.5 |
| **IUGR_5** | 91.4 | 96.4 | 101.4 | 48.0 | 69.1 | 46.7 | 122.2 | 85.5 |
| **IUGR_6** | 84.2 | 98.2 | 132.4 | 69.5 | 61.4 | 113.5 | 115.4 | 49.9 |
| **IUGR_7** | 102.3 | 129.8 | 143.8 | 121.4 | 98.5 | 111.4 | 180.4 | 119.6 |
| **IUGR_8** | 84.2 | 49.8 | 111.5 | 128.9 | 60.4 | 146.5 | 91.4 | 33.7 |
| **IUGR_9** | 101.0 | 117.8 | 98.4 | 101.2 | 70.9 | 99.8 | 111.9 | 234.6 |
| **IUGR_10** | 93.8 | 87.3 | 79.1 | 127.9 | 64.7 | 104.3 | 104.7 | 225.5 |
| **IUGR_11** | 83.2 | 107.7 | 98.7 | 63.9 | 73.1 | 97.9 | 111.9 | 236.5 |
| **IUGR_12** | 77.5 | 99.6 | 99.8 | 113.6 | 78.5 | 107.0 | 102.0 | 230.5 |
| **IUGR_13** | 75.7 | 95.7 | 100.4 | 73.4 | 63.9 | 91.8 | 77.1 | 246.2 |
| **IUGR_14** | 72.0 | 111.3 | 111.4 | 105.2 | 148.5 | 113.5 | 72.5 | 206.9 |
| **IUGR_Lf_1** | 70.7 | 67.4 | 75.4 | 142.5 | 95.7 | 106.2 | 111.4 | 196.3 |
| **IUGR_Lf_2** | 80.9 | 67.3 | 70.6 | 225.8 | 94.9 | 67.5 | 114.2 | 102.8 |
| **IUGR_Lf_3** | 83.8 | 74.2 | 70.2 | 51.8 | 106.3 | 77.9 | 59.9 | 90.3 |
| **IUGR_Lf_4** | 117.1 | 98.8 | 83.0 | 91.9 | 111.5 |  | 110.6 | 211.3 |
| **IUGR_Lf_5** | 115.5 | 122.0 | 117.9 | 92.1 | 92.9 | 103.2 | 170.0 | 192.9 |
| **IUGR_Lf_6** | 111.6 | 87.0 | 96.6 | 69.5 | 63.6 | 81.6 | 197.3 | 137.8 |
| **IUGR_Lf_7** | 96.1 | 92.9 | 95.1 | 51.8 | 87.5 | 111.5 | 92.2 | 96.0 |
| **IUGR_Lf_8** | 76.3 | 67.3 | 94.1 | 73.1 | 53.6 | 119.7 | 97.7 | 34.4 |
| **IUGR_Lf_9** | 96.4 | 78.7 | 114.8 | 59.6 | 69.6 | 103.9 | 120.5 | 80.1 |
| **IUGR_Lf_10** | 59.6 | 107.2 | 114.1 | 89.1 | 118.5 | 87.6 | 77.2 | 77.3 |
| **IUGR_Lf_11** | 60.6 | 65.4 | 87.3 | 72.3 | 100.6 | 106.1 | 93.4 | 130.7 |
| **IUGR_Lf_12** | 68.8 | 103.4 | 109.5 | 69.1 | 92.8 | 98.7 | 55.1 | 113.1 |
| **IUGR_Lf_13** | 64.0 | 80.1 | 104.8 | 92.9 | 72.8 | 105.7 | 101.2 | 109.5 |

Table 2: Protein optical density normalized to actin (42 kDa) quantified for each rat (from CTL, IUGR and IUGR_Lf pups) at P21 in the cortex corresponding to figure 4, low panel. Proteins were quantified using CTL group intensity average as 100%. (n=9-14 animals per group).

| **P7 Cortex** | **GLT1** | **MCT2** | **DMT1** | **CaMKIIβ** | **Leptin R** | **Fractin** |
| --- | --- | --- | --- | --- | --- | --- |
| **CT_1** | 109.5 | 120.7 | 102.7 | 102.1 | 123.6 | 128.0 |
| **CT_2** | 89.7 | 71.5 | 101.4 | 92.9 | 78.9 | 89.7 |
| **CT_3** | 100.8 | 107.8 | 95.9 | 105.0 | 97.5 | 82.2 |
| **CT_4** | 95.9 | 88.6 | 109.6 | 81.0 | 56.3 | 100.4 |
| **CT_5** | 96.1 | 85.8 | 93.8 | 103.8 | 127.0 | 86.1 |
| **CT_6** | 108.0 | 125.7 | 96.7 | 115.2 | 116.7 | 113.4 |
| **IUGR_1** | 94.8 | 87.0 | 107.6 | 93.5 | 179.5 | 119.2 |
| **IUGR_2** | 69.4 | 86.0 | 117.4 | 76.7 | 181.9 | 82.4 |
| **IUGR_3** | 57.7 | 65.8 | 102.8 | 75.6 | 105.0 | 70.0 |
| **IUGR_4** | 113.4 | 63.5 | 108.3 | 65.1 | 127.9 | 128.0 |
| **IUGR_5** | 135.7 | 74.1 | 101.5 | 93.9 | 153.2 | 98.4 |
| **IUGR_6** | 103.8 | 66.4 | 99.1 | 81.2 | 105.0 | 120.9 |
| **IUGR_Lf_1** | 103.0 | 95.4 | 99.1 | 94.3 | 246.9 | 88.3 |
| **IUGR_Lf_2** | 83.5 | 100.0 | 110.5 | 96.4 | 246.8 | 123.3 |
| **IUGR_Lf_3** | 99.9 | 66.9 | 100.7 | 105.8 | 237.1 | 127.8 |
| **IUGR_Lf_4** | 99.7 | 170.2 | 76.3 | 112.2 | 90.0 | 68.3 |
| **IUGR_Lf_5** | 108.9 | 64.4 | 65.7 | 115.6 | 64.7 | 110.4 |
| **IUGR_Lf_6** | 76.8 | 70.4 | 77.0 | 76.5 | 106.7 | 63.8 |
| **IUGR_Lf_7** | 126.8 | 78.2 | 111.5 | 68.3 | 88.4 | 116.9 |

Table 3: Protein optical density normalized to actin (42 kDa) quantified for each rat (from CTL, IUGR and IUGR_Lf pups) at P7 in the cortex corresponding to figure 5. Proteins were quantified using CTL group intensity average as 100%. (n=6-7 animals per group).

| **P7 Striatum** | **DCX** | **NeuN** | **Synapto** | **NG2** | **GFAP** | **CD68** | **Iba1** |
| --- | --- | --- | --- | --- | --- | --- | --- |
| **CT_1** | 87.3 | 117.0 | 89.1 | 70.8 | 110.8 | 133.0 | 92.0 |
| **CT_2** | 116.8 | 117.2 | 107.3 | 112.8 | 68.7 | 69.9 | 116.5 |
| **CT_3** | 95.9 | 65.7 | 103.6 | 116.4 | 120.5 | 97.0 | 91.5 |
| **CT_4** | 129.5 | 119.8 | 101.0 | 77.5 | 42.9 | 119.5 | 141.8 |
| **CT_5** | 97.9 | 79.9 | 123.2 | 122.5 | 222.8 | 81.3 | 65.3 |
| **CT_6** | 72.7 | 100.2 | 75.7 |  | 34.3 | 99.3 | 92.9 |
| **IUGR_1** | 104.0 | 99.5 | 98.9 | 70.5 | 70.2 | 121.1 | 91.1 |
| **IUGR_2** | 104.3 | 57.7 | 70.8 | 57.3 | 102.5 | 66.5 | 40.9 |
| **IUGR_3** | 108.9 | 72.9 | 100.9 | 40.2 | 26.5 | 131.8 | 53.7 |
| **IUGR_4** | 104.9 | 127.4 | 106.4 | 96.2 | 47.7 | 94.6 | 202.9 |
| **IUGR_5** | 115.2 | 73.8 | 105.7 | 109.8 | 146.4 | 58.9 | 244.5 |
| **IUGR_6** | 88.1 | 114.4 | 119.5 | 115.0 | 26.3 | 71.4 | 176.9 |
| **IUGR_Lf_1** | 113.7 | 86.0 | 78.3 | 89.6 | 57.8 | 143.8 | 102.7 |
| **IUGR_Lf_2** | 84.2 | 106.3 | 86.9 | 91.7 | 44.7 | 134.7 | 71.2 |
| **IUGR_Lf_3** | 77.5 | 105.1 | 79.3 | 55.2 | 36.6 | 74.1 | 77.5 |
| **IUGR_Lf_4** | 94.6 | 108.6 | 115.7 | 120.0 | 237.2 | 114.5 | 73.2 |
| **IUGR_Lf_5** | 141.0 | 86.0 | 91.3 | 158.8 | 86.2 | 104.4 | 115.3 |
| **IUGR_Lf_6** | 64.6 | 109.1 | 101.6 | 176.5 | 90.0 | 63.6 | 138.3 |
| **IUGR_Lf_7** | 103.6 | 110.2 | 103.6 | 114.8 | 155.1 | 97.0 | 193.0 |

Table 4: Protein optical density normalized to actin (42 kDa) quantified for each rat (from CTL, IUGR and IUGR_Lf pups) at P7 in the striatum corresponding to figure 8, high panel. Proteins were quantified using CTL group intensity average as 100%. (n=5-7 animals per group).

| **P21 Striatum** | **MCT2** | **NMDAR2a** | **DMT1** | **CaMKIIβ** | **Leptin R** | **TrkB** | **IGF2** |
| --- | --- | --- | --- | --- | --- | --- | --- |
| **CT_1** | 96.0 | 113.5 | 101.9 | 106.5 | 110.4 | 111.4 | 94.0 |
| **CT_2** | 119.0 | 94.7 | 108.0 | 95.7 | 90.4 | 79.3 | 104.4 |
| **CT_3** | 85.0 | 91.7 | 90.1 | 97.8 | 99.2 | 109.3 | 101.6 |
| **CT_4** | 104.1 | 175.5 | 104.8 | 89.8 | 128.0 | 133.1 | 88.1 |
| **CT_5** | 88.8 | 59.5 | 130.9 | 93.1 | 104.4 | 40.1 | 118.8 |
| **CT_6** | 107.1 | 65.0 | 64.3 | 117.1 | 67.6 | 126.8 | 93.1 |
| **CT_7** | 102.1 |  |  | 109.1 | 90.3 |  |  |
| **CT_8** | 97.9 |  | 100.0 | 90.9 | 109.7 |  |  |
| **CT_9** | 91.5 |  | 116.3 | 94.3 | 100.7 |  |  |
| **CT_10** | 108.5 |  | 83.7 | 105.7 | 99.3 |  |  |
| **IUGR_1** | 85.7 | 119.6 | 64.6 | 79.1 | 95.8 | 198.4 | 117.4 |
| **IUGR_2** | 80.8 | 58.3 | 44.9 | 60.7 | 78.8 | 90.5 |  |
| **IUGR_3** | 89.7 | 43.4 | 68.7 | 69.0 | 60.7 | 98.2 | 228.1 |
| **IUGR_4** | 87.8 | 82.6 | 82.0 | 74.1 | 82.4 | 39.4 | 160.4 |
| **IUGR_5** | 113.9 |  | 97.7 | 78.7 | 73.8 |  |  |
| **IUGR_6** | 95.7 |  | 72.0 | 79.3 | 118.2 |  |  |
| **IUGR_7** | 113.2 |  | 119.6 | 62.7 | 101.2 |  |  |
| **IUGR_8** | 116.5 |  | 85.9 | 66.2 | 96.1 |  |  |
| **IUGR_9** | 109.8 |  | 101.0 | 102.7 | 119.5 |  |  |
| **IUGR_10** | 104.1 |  | 98.6 | 96.4 | 92.9 |  |  |
| **IUGR_11** | 107.6 |  | 99.7 | 91.4 | 65.4 |  |  |
| **IUGR_12** | 119.8 |  | 93.4 | 97.3 | 92.8 |  |  |
| **IUGR_13** | 119.4 |  | 104.3 | 77.5 | 30.3 |  |  |
| **IUGR_14** | 125.2 |  | 63.9 | 90.1 | 82.3 |  |  |
| **IUGR_Lf_1** | 102.0 | 65.0 | 95.5 | 75.6 | 72.3 | 124.5 | 143.2 |
| **IUGR_Lf_2** | 84.4 | 102.1 | 75.5 | 84.5 | 86.4 | 74.6 | 135.4 |
| **IUGR_Lf_3** | 90.8 | 33.6 | 63.0 | 79.3 | 38.5 | 34.2 |  |
| **IUGR_Lf_4** | 65.1 | 17.4 | 61.3 | 69.6 | 55.6 | 18.8 | 129.7 |
| **IUGR_Lf_5** | 124.5 |  | 106.8 | 82.4 | 113.3 |  |  |
| **IUGR_Lf_6** | 110.6 |  | 95.4 | 82.9 | 126.3 |  |  |
| **IUGR_Lf_7** | 110.4 |  | 70.7 | 83.3 | 44.2 |  |  |
| **IUGR_Lf_8** | 115.6 |  | 106.5 | 81.5 | 89.6 |  |  |
| **IUGR_Lf_9** | 115.6 |  | 115.0 | 86.2 | 67.6 |  |  |
| **IUGR_Lf_10** | 119.4 |  | 106.3 | 101.7 | 92.7 |  |  |
| **IUGR_Lf_11** | 90.9 |  | 94.3 | 90.3 | 74.3 |  |  |
| **IUGR_Lf_12** | 93.7 |  | 103.2 | 84.6 | 112.0 |  |  |
| **IUGR_Lf_13** | 95.1 |  | 102.8 | 95.3 | 84.6 |  |  |

Table 5: Protein optical density normalized to actin (42 kDa) quantified for each rat (from CTL, IUGR and IUGR_Lf pups) at P21 in the striatum corresponding to figure 8, low panel. Proteins were quantified using CTL group intensity average as 100%. (n=3-14 animals per group).
